# Clinical outcome and genomic biomarkers of immune checkpoint inhibitor-based therapies for cancer of unknown primary: a multicenter, real-world study

**DOI:** 10.1007/s00432-025-06261-3

**Published:** 2025-07-12

**Authors:** Yunjie Huang, Riqing Huang, Meiting Chen, Zhousan Zheng, Haifeng Li, Rishang Chen, Tinghua Gao, Ditian Shu, Anqi Hu, Qiufan Zheng, Xin An, Yanxia Shi, Cong Xue

**Affiliations:** 1 State Key Laboratory of Oncology in South China, Guangdong Provincial Clinical Research Center for Cancer, Sun Yat-Sen University Cancer Center, Guangzhou, 510060 People’s Republic of China; 2 Department of Medical Oncology, State Key Laboratory of Oncology in South China, Sun Yat-Sen University Cancer Center, Dongfeng Road East 651, Guangzhou, 510060 People’s Republic of China; 3 Department of Oncology, The First Affiliated Hospital of Sun Yat-Sen University, No. 58, Zhongshan Road II, Guangzhou, 510080 People’s Republic of China; 4 Medical Oncology Department III, Central Hospital of Guangdong Nongken, Zhanjiang, 524002 China; 5 Department of Oncology, The First People’s Hospital of Zhaoqing City, Zhaoqing, Guangdong China

**Keywords:** Cancer of unknown primary, Programmed death-ligand 1 expression, KRAS, Immune checkpoint inhibitor, Genomic biomarker

## Abstract

**Background:**

Given the limited treatment options recommended for cancer of unknown primary (CUP), especially the role of immune checkpoint inhibitors (ICIs), our study aimed to evaluate the efficacy of ICIs and identify associated genomic biomarkers in these patients.

**Methods:**

This retrospective, multicenter, real-world analysis included individuals with oncologist-confirmed CUP cases treated with ICIs across four hospitals in China. Clinical outcomes, safety and biomarkers were analyzed.

**Results:**

Between January 2016 and November 2023, 124 patients were enrolled. Of these, 117 patients underwent combination therapy, predominantly ICIs with taxane-platinum based chemotherapy (54.84%), while 7 received monotherapy. After a median follow-up of 18.6 months, the median progression free survival (PFS) and overall survival (OS) were 23.20 and 38.86 months, respectively. According to ESMO guideline, patients were stratified into favorable (n = 41) and unfavorable subset (n = 83). The favorable subset demonstrated significantly longer PFS and OS than the unfavorable subset (median PFS NR vs. 9.7 months, *P* < 0.001; median OS NR vs. 23.73 months, *P* < 0.001). There are 31 patients in our cohort whose PD-L1 detection results are available, while 25 patients was eligible for TMB assessment. Better clinical efficacy of ICIs was apparent for tumors with a higher PD-L1 expression (CPS ≥ 20) and a greater tumor mutation burden (> 12 mutations/Mb). Multivariate analyses revealed that higher ECOG performance status, the presence of visceral metastasis, and *KRAS* mutation were independently associated with inferior PFS and OS. Immune-related adverse events occurred in 49 (39.52%) patients, with one developing grade 3 pneumonia.

**Conclusions:**

The application of ICIs showed encouraging efficacy with an acceptable safety profile, suggesting its potential as an additional therapeutic option for CUP patients. Identifying predictive markers for ICIs response remains essential to enhance therapeutic strategies in CUP management.

**Supplementary Information:**

The online version contains supplementary material available at 10.1007/s00432-025-06261-3.

## Introduction

Cancer of unknown primary (CUP) is defined as histologically confirmed metastatic cancer for which the primary site is unidentifiable by standardized clinical and pathological workup (Krämer et al. 2023). These heterogeneous tumors constitute < 5% of all diagnosed cancers (Brewster et al. 2014; Pavlidis and Pentheroudakis 2012; Siegel et al. 2023) and present with diverse clinical manifestations, ranging from solitary sites of disease to multiple metastases (Krämer et al. 2023). Affected sites commonly include lymph nodes, bone, liver, and lung (Abbruzzese et al. 1994; Kolling et al. 2019). Notoriously, CUP patients have a poor prognosis, with a median survival time of less than 12 months (Pavlidis and Pentheroudakis 2012; Kang et al. 2021; Golfinopoulos et al. 2009). Early dissemination, aggressiveness, and unpredictable metastatic patterns are characteristic of these tumors (Pavlidis and Pentheroudakis 2012). Challenge arises due to the unidentifiable primary site of cancer, significantly impacting clinical management, as determining the primary tumor site is pivotal in guiding treatment decisions and overall prognosis for patients (Randén et al. 2009).

According to the European Society of Medical Oncology (ESMO) classification, CUP can be stratified into favorable and unfavorable subgroups (Krämer et al. 2023). Favorable CUP is characterized by histological similarities to cancers with known primaries, like those of the colon, the breast, the ovary, and the prostate, where established systemic therapies are available, guiding effective treatment approaches. Approximately 20% of patients with CUP have favorable prognostic factors, respond to specific local therapy with or without systemic therapy, and typically have a median overall survival (OS) of 12–36 months (Pavlidis and Pentheroudakis 2012). Conversely, most patients with CUP are categorized into an unfavorable subset and receive empirical chemotherapy, such as platinum- and taxane-based regimens (Briasoulis et al. 2000; Greco et al. 2000; Greco and Pavlidis 2009), while the survival benefit for these regimens is about 6 to 12 months (Greco and Pavlidis 2009; Petrakis et al. 2013; Pavlidis et al. 2015; Lee et al. 2013). Subsequent lines of chemotherapy yield a median progression-free survival (PFS) and OS of only 2–4 and 3–9 months, respectively (Culine et al. 2001; Hainsworth et al. 2001; Hainsworth et al. 2010; Hainsworth et al. 2007; Hainsworth et al. 2005; Møller et al. 2010). The exploration of personalized medicine, including gene expression-guided chemotherapy or genome sequence-guided molecular therapy, has shown promise. However, while approximately 30% of unfavorable CUPs display genomic features hinting at targeted therapy potential (Ross et al. 2015; Westphalen et al. 2023), their clinical benefit from these emerging therapies remains unclear (Ross et al. 2015; Hainsworth et al. 2013; Hayashi et al. 2019). Given its heterogeneous and aggressive nature, the treatment of CUP is problematic and not well developed.

In the current landscape, immune checkpoint inhibitors (ICIs) represent a promising therapeutic strategy that has led to a marked improvement in survival for patients with various types of malignancy. Realizing substantial improvements in CUP patient outcomes demands access to more effective immunotherapies. A retrospective study utilizing a 92-gene assay (CancerTYPE ID) found that 38% of CUPs were categorized as cancer types approved for access to ICI-based therapies by the US Food and Drug Administration (FDA) (Raghav et al. 2020). Additionally, 28% of CUP tumors harbored one or more predictive biomarkers, such as microsatellite instability-high (MSI-H), Programmed death ligand-1 (PD-L1) positive and/or tumor mutation burden high (TMB-H), associating with the immune checkpoint blockade (Gatalica et al. 2018). Moreover, a notable fraction of CUP tumors exhibited heavy T- cell infiltrates or expressed T- cell inflammatory markers, akin to ICI-responsive malignancies (Haratani et al. 2019). Hence, ICIs could potentially emerge as a viable treatment for CUP.

The application of ICIs has expanded rapidly in clinical trials and real-world settings, attributed to their potentially robust therapeutic responses. Phase II trials indicated pembrolizumab and nivolumab in refractory CUP achieved overall response rates (ORRs) of 23% and 24%, respectively, while the nivolumab and ipilimumab combination therapy yielded a 16% ORR (Pouyiourou et al. 2023; Raghav, et al. 2022; Tanizaki et al. 2022). The median PFS of these three trials ranged from 2.5 to 4.1 months, while the OS varied between 3.8 and 15.9 months. However, the sample sizes of these studies are relatively small, with the sample size eligible for analysis fewer than 50 cases, making them less likely to adequately represent the broader population or account for potential confounding variables. On the other hand, previous prospective trials indicated that enrolling over 100 patients with CUP can take 3–6 years (Hayashi et al. 2019; Hayashi et al. 2020), indicating the difficulty of conducting large trials for rare cancers. Thus, in such situations, the analysis of real-world data becomes crucial to further support the clinical trial results. Additionally, Previous studies predominately included CUP patients who had received at least 1 line of chemotherapy before enrollment, and the patients are often treated with ICIs monotherapy (Pouyiourou et al. 2023; Raghav, et al. 2022; Tanizaki et al. 2022). In our cohort, a considerable proportion of CUP patients received ICIs combined with chemotherapy as first-line treatment. Therefore, we initiated a multicenter, real-world retrospective study to evaluate the efficacy of ICIs and explore its genomic biomarkers of CUP.

## Methods

### Study design and patients

This retrospective, multicenter, real-world study included patients with CUP who received ICIs at Sun Yat-Sen University Cancer Centre (SYSUCC), the First Affiliated Hospital of Sun Yat-Sen University, Central Hospital of Guangdong Nongken and the First People's Hospital of Zhaoqing City between January 2016 and November 2023. The study protocol was approved by the ethical committee of SYSUCC (approval number B2023-684-01). Eligible patients were ≥ 18 years old and met clinical and histologic criteria for CUP, received at least one dose of ICIs with available response assessment, and had adequate cardiac, bone marrow, and hepatic function apart from organ function affected by the disease. All cases had comprehensive clinical documentation, and primary lesions were not detected through various diagnostic tools such as x-rays, computed tomography (CT), MRI, PET/CT, and endoscopy. Finally, all initially screened patients were reviewed by SYSUCC oncologists and pathologists to select CUP patients. The classification of patients into favorable and unfavorable subsets was based on published ESMO guidelines (Krämer et al. 2023). The requirement for individual informed consent was waived by the committee because of the retrospective nature of the study.

The data were extracted from the medical charts and included the patients’ demographics, tumor characteristics, treatment, standard laboratory tests, and image scans. The patients’ treating physicians decided regimens, and the information was recorded. All patients in this study received at least one dose of ICIs, including Pembrolizumab, Nivolumab, Atezolizumab, Durvalumab, Sintilimab, Toripalimab, and Tislelizumab, Camrelizumab, Serplulimab. The strategy of PD-1 antibody was decided by experienced oncologists and administered based on instructions.

### Assessment of safety and treatment response

Adverse events (AEs) were graded according to the Common Terminology Criteria for Adverse Events version 5.0. The relative frequency of each AE considered possibly, probably, or likely related to chemotherapy or ICIs was estimated as the proportion of all toxicity-evaluable cycles in which toxicity was observed. The safety analysis set included all the patients who received at least one dose of the study treatment.

*The* objective response was sustained for a minimum of two consecutive imaging evaluations at least four weeks apart. The disease was also evaluated using RECIST version 1.1 for response assessment. Survival was measured from initiation of therapy until death. The disease control rate (DCR), ORR, PFS, and OS were also analyzed. OS and PFS were calculated from the start of ICIs to death, and to progression or death, respectively. The DCR was calculated as the proportion of patients achieving a complete response (CR), partial response (PR), or stable disease (SD). The ORR was calculated as the proportion of patients achieving a CR or a PR. The duration of response (DOR) was defined as the time from the first evaluation of CR, PR, or SD to PD. Time to response was defined as the time to first response.

### PD-L1 expression

PD-L1 expression on tumor and mononuclear inflammatory cells in tumor nests were assessed at our institution's pathology department by immunohistochemistry using Merck 22C3 antibody. The combined positive score (CPS) for PD-L1 expression was also calculated as the number of PD-L1-positive cells (tumor cells, lymphocytes, macrophages) divided by the total number of tumor cells and multiplied by 100 (Ott et al. 2019). Immunohistochemistry stainings were evaluated by a specialist in pathology from our institution's pathology department and scoring of PD-L1 was performed according to standardized scoring criteria. PD-L1 positivity was defined as CPS ≥ 1.

### Targeted sequencing

Genomic testing encompassed hybrid capture-based targeted next-generation sequencing (NGS) on the Illumina sequencing platform to identify targetable alterations. Initially, DNA was extracted and sheared from archival formalin-fixed paraffin-embedded tumor along with matched normal tissues. Subsequently, sequencing libraries were generated, ensuring a consistent median depth (> 500 ×), and assessed for somatic variants, including single nucleotide variants, small insertions and deletions, copy number alterations, and gene fusions/rearrangements. A cut-off of 12 mutations per megabase was established to classify patients into high and low TMB groups, based on the cut-off value derived from a phase II trial of nivolumab and ipilimumab in patients with recurrent or refractory cancer of unknown primary (Pouyiourou et al. 2023). The cut-off value of 12 mutations/Mb is slightly higher than the 10 mutations/Mb approved by the FDA to ensure greater specificity. A previous study which included 7,033 samples demonstrated that the cutpoint of the highest 20% of the TMB in CUP patients is higher than those in most other solid tumors (Samstein et al. 2019). Therefore, we set a cut-off value of 12 mutations/Mb to further identify a subgroup of CUP patients who are more likely to have a better response to ICIs.

### Statistical analysis

Statistical analyses and data visualization were performed using R version 4.2.2 (The R Project for Statistical Computing, www.r-project.org). A cut-off date of December 26th, 2023 was established for analyzing data for this report. The Kaplan–Meier method was used to estimate PFS and OS, and differences were evaluated by the log-rank test. Given the small sample size of patients who underwent next-generation sequencing and TMB assessment (n = 26 and n = 25), Fisher’s exact test was used to compare response rates instead of the chi-square test, as the expected frequencies in contingency tables were below 5. Multivariate analysis of survival data was conducted using the Cox proportional hazard model according to prespecified stratification factors to determine the prognostic value of variables. Incidence and severity of adverse events were analyzed for the safety population. Two-sided*P * < 0.05 were considered statistically significant.

## Results

### Patient characteristics

Our cohort consisted of 124 patients with complete clinical profiles and follow-up data. Among them, 117 underwent combination therapy, with 68 (54.84%) receiving ICIs with taxane-platinum based chemotherapy (ICIs + TP) and 49 (39.52%) being administered ICIs with other chemotherapy (ICIs + other), while 7 (5.65%) received ICIs monotherapy. The patients in our cohort were 18 to 80 years of age, with 44 patients (35.48%) aged more than 60 years old. Most patients were male (72.58%), and 31.45% of them had a history of smoking. Other patient and disease characteristics are summarized in Table 1. To identify the primary tumor, 84.68% of them underwent PET/CT, and one patient underwent PET/MR, while the remaining patients were mainly diagnosed through CT, MRI, and endoscopy. Patients received ICIs approved by the FDA or National Medical Products Administration of China, including sintilimab (25.81%), pembrolizumab (21.77%), and tislelizumab (17.74%). The details of the combination regimens are provided in Table S1. Regarding local treatments post-ICIs, 6 patients underwent surgery, and another 28 received radiotherapy.

**Table 1:** Baseline characteristics

	Patients (N = 124), N (%)
Age	
Median (range)	55 (18–80)
Sex	
Female	34 (27.42)
Male	90 (72.58)
ECOG performance status	
0	82 (66.13)
1	38 (30.65)
2	4 (3.23)
Smoking history	39 (31.45)
Histology	
Adenocarcinoma	42 (33.87)
Squamous cell carcinoma	54 (43.55)
Undifferentiated carcinoma	19 (15.32)
Other	9 (7.26)
CUP subtype	
Favorable subset	41 (33.06)
Unfavorable subset	83 (66.94)
Visceral metastasis site	
Peritoneal or omental implantation	16 (12.90)
Adrenal gland	8 (6.45)
Liver	12 (9.68)
Lung	14 (11.29)
Bone	24 (19.35)
Brain	3 (2.42)
Prior radiotherapy	19 (15.32)
Prior surgery	29 (23.39)
Treatment line	
1st	81 (65.32)
≥ 2nd	43 (34.68)
Combination therapy	
ICIs + TP based chemotherapy	68 (54.84)
ICIs + Other	49 (39.52)
Monotherapy	7 (5.65)

^*^ECOG, Eastern Cooperative Oncology Group; TP, taxane-platinum

### Efficacy outcome

**Fig. 1:**
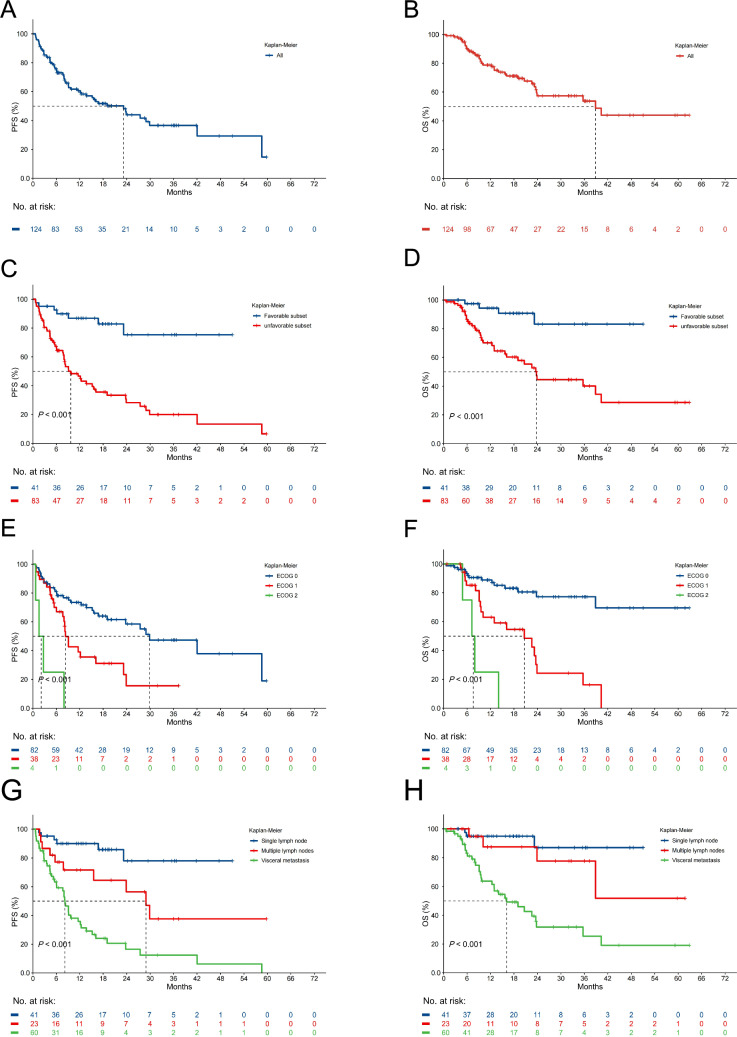
Clinical efficacy of ICIs. Kaplan–Meier plots for **A** progression-free survival (PFS) and **B** overall survival (OS) for all patients. Kaplan–Meier plots for **C** PFS and **D** OS according to CUP subtype. Kaplan–Meier plots for **E** PFS and **F** OS according to ECOG performance status. Kaplan–Meier plots for **G** PFS and **H** OS according to metastatic sites

At the data cut-off for this analysis, the median follow-up duration was 18.6 months (95% confidence interval [CI] 16.97–23.63). Among all patients, 19 (15.32%) individuals had a confirmed CR and 50 (40.32%) had a confirmed PR, yielding an ORR of 55.65%. Furthermore, the ORR was 80.49% in the favorable subset, compared to 43.37% in the unfavorable subset (Table S2). Treatment was ongoing in twenty patients (16.13%). Notably, 16 individuals (12.9%) maintained ICIs for more than 1 year, with 3 were continuing for 2 years. Although 60 patients experienced progression on the treatment, 25 of them continued ICIs beyond progression. Among all included patients, the one-year PFS and OS rates were 59.1% and 77.6%, respectively. The median PFS was 23.20 (95% CI 12.37—not reach[NR]) months, while the median OS was 38.86 (95% CI 23.73—NR) months (Fig. 1A, B). Both PFS and OS were significantly longer in patients within the favorable subset compared to the unfavorable subset (median PFS NR vs. 9.7 months, *P* < 0.001; median OS NR vs. 23.73 months, *P* < 0.001; Fig. 1C, D). Notably, lower ECOG status was substantially associated with superior PFS and OS (median PFS 29.87 vs. 8.33 vs. 2.13 months, *P* < 0.001; median OS NR vs. 20.67 vs. 7.60 months, *P* < 0.001; Fig. 1E, F). Additionally, the presence of visceral metastasis demonstrated a higher risk for progression and death in comparison to multiple lymph nodes metastasis or single lymph node metastasis (median PFS 8.2 vs. 28.93 months vs. NR, *P* < 0.001; median OS 16.13 months vs. NR vs. NR, *P* < 0.001; Fig. 1G, H). Moreover, first-line ICIs treatment resulted in better PFS and OS than ≥ 2nd line (median PFS NR vs. 11.6 months, *P* = 0.002; median OS NR vs. 23.73 months, *P* = 0.045; Fig. S1**A, B**).

**Fig. 2:**
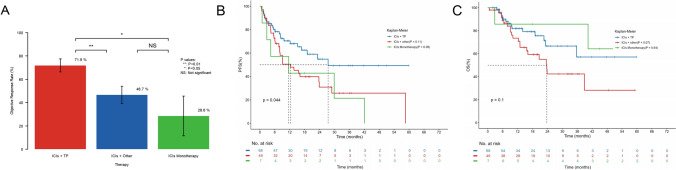
Clinical efficacy of patients receiving different combination therapies. ORR by combination therapy **A** Kaplan–Meier plots for **B** PFS and **C** OS according to combination therapy

Patients receiving ICIs + TP showed significantly higher ORR than those receiving ICIs + other (71.9% vs. 46.7%; *P* = 0.0097; Fig. 2A) and ICIs monotherapy (71.9% vs. 28.6%; *P* = 0.032; Fig. 2A). Additionally, patients who received ICIs + TP had longer PFS compared to ICIs + other (27.4 vs. 12.4 months, HR = 0.55, *P* = 0.11; Fig. 2B) and ICIs monotherapy (27.4 vs. 11.6 months, HR = 0.44, *P* = 0.08; Fig. 2B), while the OS of patients receiving ICIs + TP is also longer than those receiving ICIs + other (NR vs. 23.7 months, HR = 0.54, *P* = 0.07; Fig. 2C), though they did not reach statistical significance.

### Biomarker analyses

**Fig. 3:**
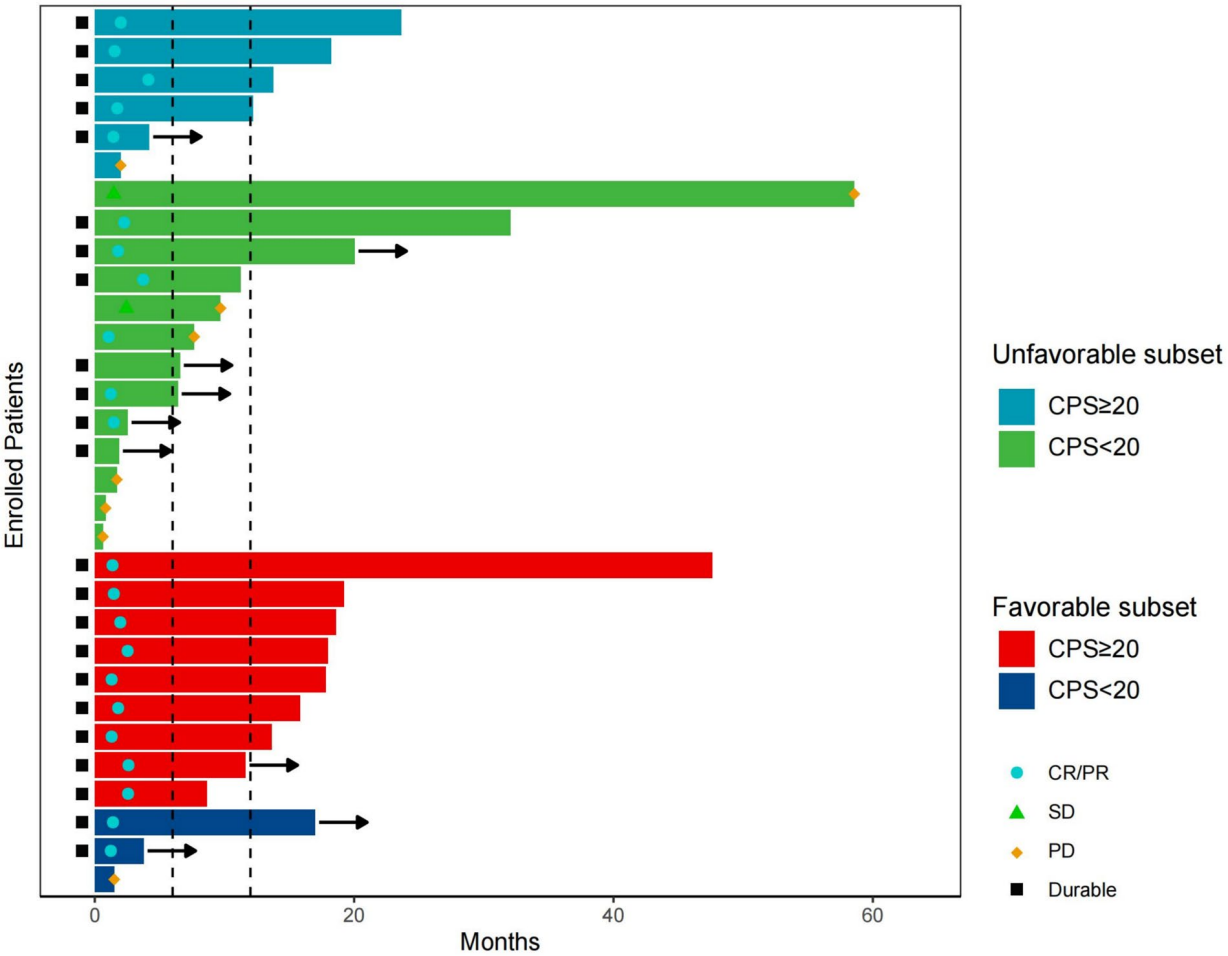
Swimmer plot of the patients with available data of different PD-L1 expressions. Arrows indicate patients still on treatment

Higher PD-L1 expression (CPS ≥ 20) was found to be associated with better long-term responses, particularly in unfavorable cases. In 31 patients with available PD-L1 detection results, the positivity rate for PD-L1 (CPS ≥ 1) was 83.87%, and 10 patients (32.26%) had CPS ≥ 50. Among these patients, 19 were classified as unfavorable cases, while 12 were favorable cases. Other disease characteristics of these patients are presented in Table S3. CPS ≥ 20 was selected as the cutoff for biomarker analysis based on a prior study (Burtness et al. 2019) and its proximity to the median CPS in our cohort. In the unfavorable group, for those with PD-L1 CPS ≥ 20 (N = 6), 4 patients (66.67%) had responses lasting over 12 months, with only 1 patient (16.67%) experiencing disease progression; Conversely, among those with PD-L1 CPS < 20 (N = 13), only 3 patients (23.08%) had responses lasting over 12 months, while 5 patients (38.46%) experienced disease progression. Similarly, in the favorable group, for patients with PD-L1 CPS ≥ 20 (N = 9), 7 (77.78%) had responses lasting over 12 months, with no cases of disease progression. Among those with PD-L1 CPS < 20 (N = 3), only 1 patient had a response lasting over 12 months, while 1 patient experienced disease progression (Fig. 3).

**Fig. 4:**
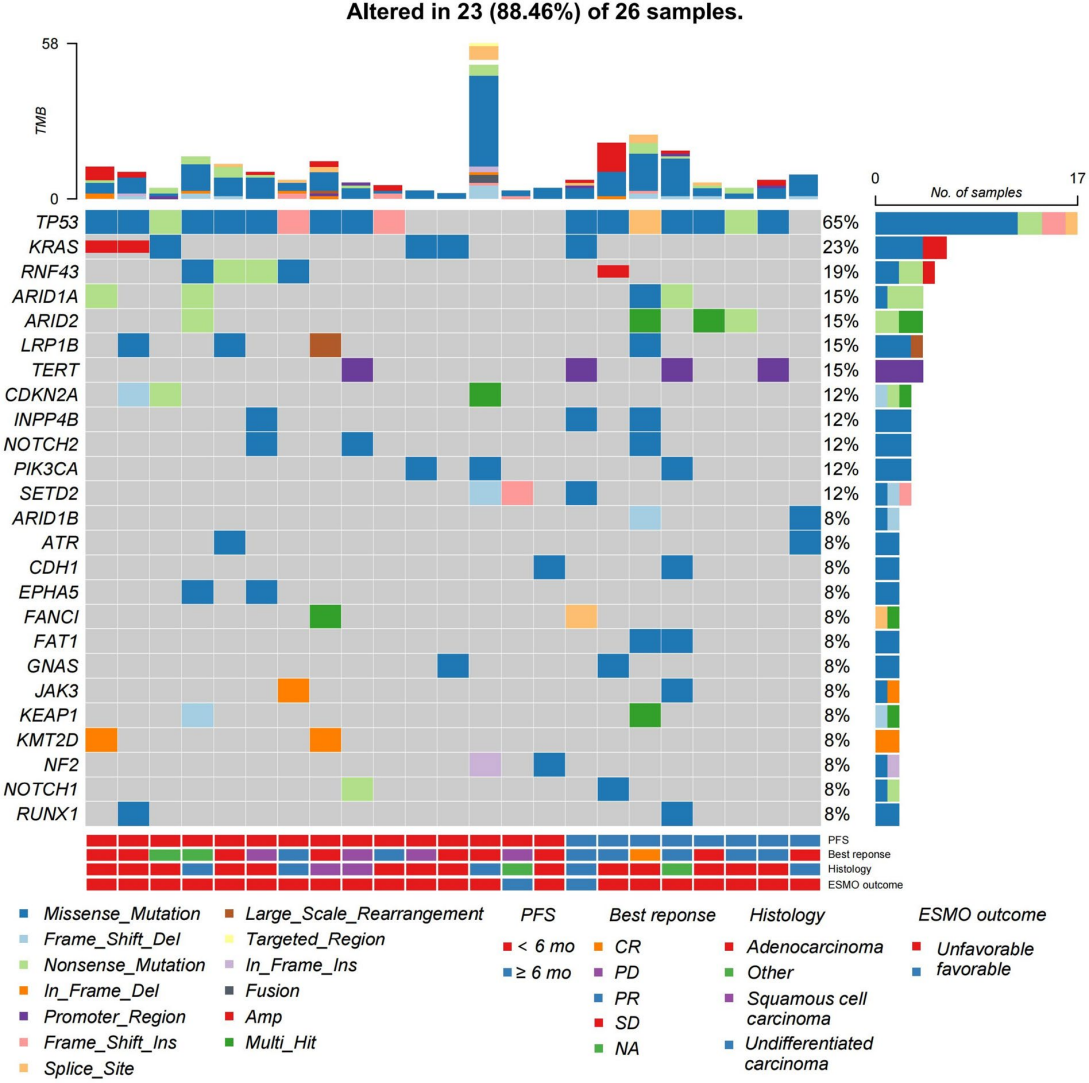
The landscape of high-frequency altered genes in patients with CUP. Each column represents an individual patient, while rows denote various genes or genetic loci analyzed

**Fig. 5:**
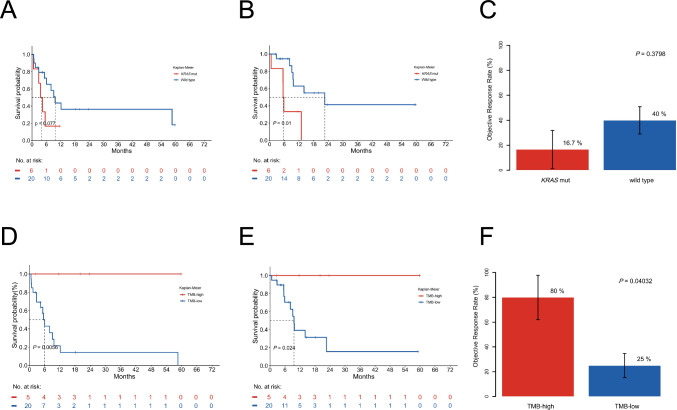
Patients with *KRAS* mutation did not benefit from ICI treatment: Kaplan Meier estimates of **A** PFS and **B** OS, stratified according to *KRAS* mutation. **C** ORR by *KRAS* mutation. High tumor mutation burden (TMB) is predictive of clinical outcome: Kaplan Meier estimates of **D** progression-free survival (PFS) and **E** overall survival (OS), stratified according to TMB. **F** Objective response rate (ORR) by TMB

We performed next-generation sequencing on 26 patients and observed that CUP patients harboring *KRAS* alterations did not demonstrate significant benefits from ICI treatment. Among the 26 patients, 3 were favorable cases, while the rest were unfavorable cases. Disease characteristics of these patients are presented in Table S4. The Oncoplot graphically illustrated the mutational landscape and distribution of genetic alterations within these patients (Fig. 4). Among the detected abnormalities, the top three in terms of mutation frequencies were *TP53* alterations (65%), *KRAS* mutations (23%), and *RNF43* alterations (19%), respectively. None of the samples showed MSI. Noteworthy, patients with a PFS < 6 months exhibited a higher incidence of *KRAS* mutations (33.33%) compared to those with a PFS ≥ 6 months (9.1%). Patients with *KRAS* mutation had shorter PFS (3.96 vs. 9.7 months, HR = 2.63, *P* = 0.077; Fig. 5A) and significantly lower OS (5.68 vs. 22.6 months, HR = 4.48, *P* = 0.019; Fig. 5B) compared to wild-type. Additionally, individuals with *KRAS* mutations exhibited lower ORR than those without, though not statistically significant (Fig. 5C).

Of the 26 patients who underwent next-generation sequencing, 25 patients' samples were eligible for TMB assessment. Our analysis revealed that high TMB (≥ 12 mutations/Mb) was associated with better treatment efficacy and survival outcome. Among these 25 CUP patients, 5 patients were stratified to be TMB-H and 20 patients to be TMB-low (TMB-L), based on a TMB cut-off of 12 mutations/Mb. The mean TMB across the CUP cohort was 6.96 mutations/Mb, with a median of 5.33 mutations/Mb. Survival analysis revealed significantly longer PFS and OS in the TMB-H group. Specifically, the one-year PFS rate was 100% for the TMB-H group, compared to 14.3% for the TMB-L cohort, with a median PFS of NR versus 6.03 months (*P* = 0.006), respectively (Fig. 5D). Similarly, the one-year OS rates of TMB-H and TMB-L cohorts were 100% and 31.3%, respectively, with the median OS not reached in the TMB-H group and 9.57 months in the TMB-L patients (*P* = 0.024; Fig. 5E). Moreover, the TMB-H group were found to have a better ORR than the TMB-L group (80% vs. 25%; *P* = 0.040; Fig. 5F).

### Correlative analyses

**Fig. 6:**
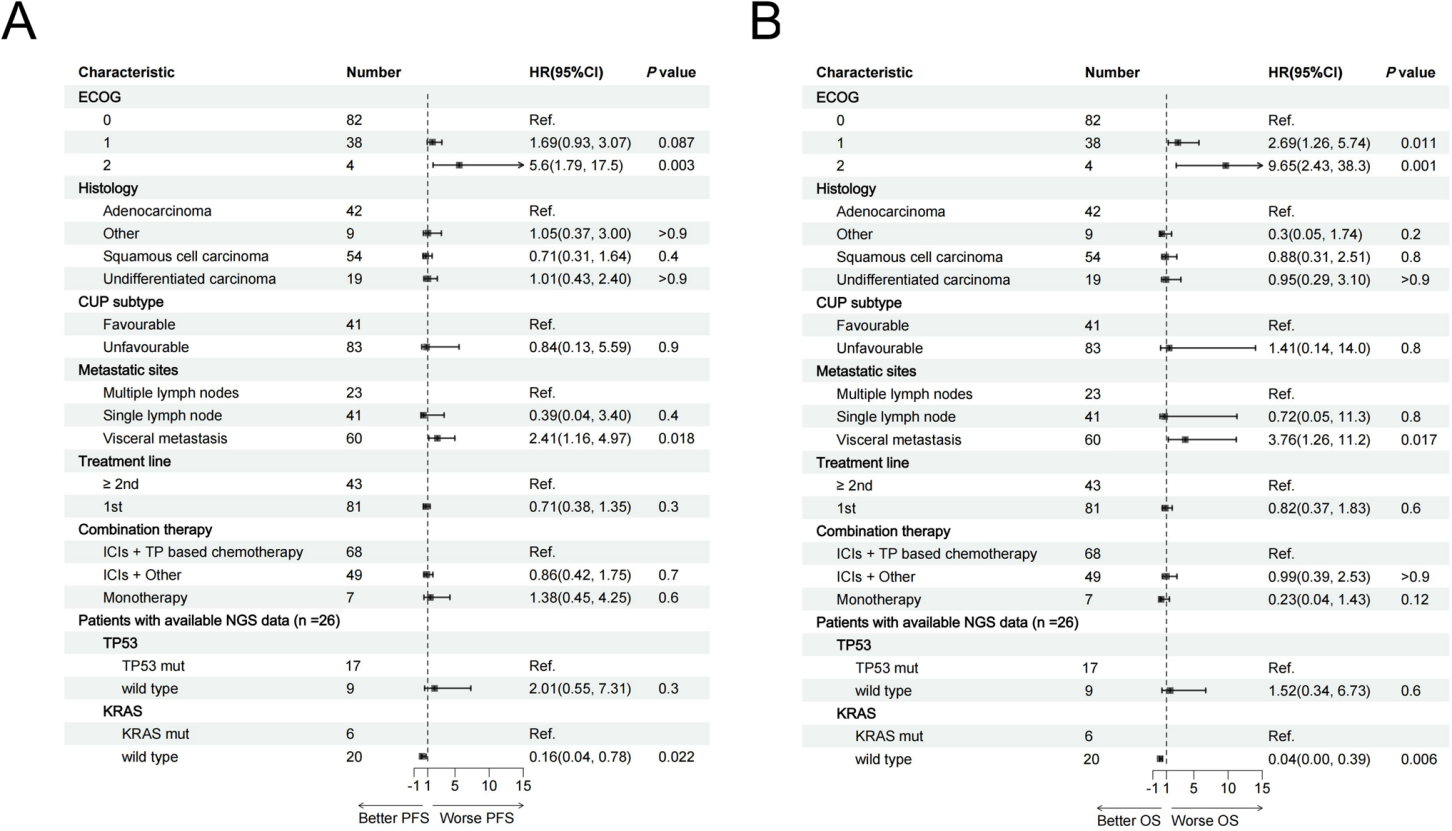
Forest plot for **A** progression-free survival and **B** overall survival according to prespecified subgroups in multivariable Cox regression analysis. Ref., referencer. HR, hazard ratio. CI, confidence interval

To assess the factors that affect the efficacy of ICIs, we conducted comprehensive univariable and multivariate analyses investigating the correlation between clinicopathologic factors, oncogenic drivers, and patient outcomes (Fig. 6; Fig S2). The results indicated that higher ECOG performance status, the presence of visceral metastasis, and *KRAS* mutation were independently associated with inferior PFS and OS within our patient cohort. After adjusting for various variables, higher ECOG status exhibited a notable impact on PFS (HR = 5.6, 95% CI 1.79–17.5, *P* = 0.003), and OS (HR = 9.65, 95% CI 2.43- 38.3, *P* = 0.001). Similarly, visceral metastasis emerged as an independent predictor for inferior PFS (HR = 2.41, 95% CI 1.16–4.97, *P* = 0.018) and OS (HR = 3.76, 95% CI 1.26–11.2, *P* = 0.017) across both univariable and multivariate analyses. Notably, among CUP patients with accessible *KRAS* mutation status, individuals with *KRAS* wild type remained a significant association with both PFS (HR = 0.16, 95% CI 0.04–0.78, *P* = 0.022) and OS (HR = 0.04, 95% CI 0.00–39, *P* = 0.006) even after adjustments when compared to cases harboring KRAS mutations. However, the impact of CUP subtype and treatment line on PFS and OS did not maintain significance in the multivariate model.

### Safety

Adverse events were consistent with the known safety profile of ICIs. The incidences of any AEs and grade 3 to 4 AEs in all patients are summarized in Table 2. Among the cohort, 121 (97.58%) individuals experienced AEs of any grade, with 27 (21.77%) encountering AEs of grade 3 or 4. Serious AEs were observed in 11 (8.87%) patients, but no treatment-related death occurred. The most common AEs of any grade that occurred after the start of treatment consisted of anemia (72.58%), leukopenia (51.61%), neutropenia (44.35%), dyspepsia (39.52%), and elevated transaminases (37.10%). Immune-related AEs occurred in 49 (39.52%) patients, with most events being of grade 1 or 2. The most prevalent immune-related toxicities comprised hypothyroidism (12.10%), pruritus (12.10%), and rash (9.68%). Notably, One patient developed grade 3 immune-related pneumonia and required treatment discontinuation.

**Table 2:** Incidence of adverse events (AEs) for all patients (N = 124)

Event	Cases, N (%)	
	Any Grade	Grade 3 or 4
Any AE	121 (97.58)	27 (21.77)
Leukopenia	64 (51.61)	10 (8.06)
Neutropenia	55 (44.35)	9 (7.26)
Febrile neutropenia	4 (3.23)	2 (1.61)
Thrombocytopenia	31 (25.00)	12 (9.68)
Anemia	90 (72.58)	13 (10.48)
Serum creatinine increased	23 (18.55)	0
Elevated transaminases	46 (37.10)	1 (0.81)
Fatigue	39 (31.45)	0
Dyspepsia	49 (39.52)	0
Nausea	42 (33.87)	0
Vomiting	27 (21.77)	0
Diarrhea	4 (3.23)	0
Constipation	19 (15.32)	1 (0.81)
Peripheral neuropathy	15 (12.10)	0
Hand foot syndrome	3 (2.42)	0
Immune related AEs		
Any irAE	49 (39.52)	1 (1.61)
Rash	12 (9.68)	0
Pruritus	15 (12.10)	0
Hypothyroidism	25 (20.16)	0
Hyperthyroidism	8 (6.45)	0
Adrenal_insufficiency	3 (2.42)	0
Hepatitis	2 (1.61)	0
Pneumonia	3 (2.42)	1 (1.61)

In summary, the median PFS and OS of our patients were 23.20 and 38.86 months, respectively, with an ORR of 55.65%. The patients receiving ICIs + TP showed significantly higher ORR and a non-significant trend toward improved PFS and OS than those receiving ICIs + other and ICIs monotherapy. Analyses of the 31 patients whose PD-L1 detection results were available and the 25 patients who were eligible for TMB assessment indicated better clinical efficacy of ICIs for tumors with a higher PD-L1 expression (CPS ≥ 20) and a greater tumor mutation burden (> 12 mutations/Mb). In multivariate analyses, it was revealed that higher ECOG performance status, the presence of visceral metastasis, and *KRAS* mutation were independently associated with inferior PFS and OS. The adverse events were manageable and consistent with the known safety profile of ICIs.

## Discussion

To our knowledge, this study was the first and largest multicenter real-world investigation that presents a remarkable clinical response to ICIs in a series of patients with CUP. Our study included rigorous monitoring of immune-related adverse events and revealed that these treatments were well-tolerated by patients, consistent with earlier observations from clinical trials, without any new safety concerns beyond the established toxicity profile. These insights hold significant value in guiding clinical decision-making regarding the optimal utilization of ICIs in CUP management, potentially reshaping the therapeutic approach for CUP patients.

The unique features and heterogeneity of CUP make it difficult to establish a universal standard regimen. Although the role of ICIs in CUP is evolving, there is currently no approved immunotherapeutic regimen by FDA mainly for this patient group. According to ESMO guidelines, ICIs is recommended specifically for patients with MSI-H, mismatch repair-deficient (dMMR), TMB-H, or PD-L1-high status (Krämer et al. 2023). Given the limited effective treatments available for many CUP patients, there exists a compelling rationale to utilize ICIs due to their pancancer efficacy. Our real-world analysis examining the efficacy of ICIs in CUP patients has revealed outcomes that surpass those reported in clinical trials (Pouyiourou et al. 2023; Raghav, et al. 2022; Tanizaki et al. 2022). This disparity in efficacy could potentially be attributed to several factors observed in our study. Notably, a substantial proportion of patients received ICIs as a first-line treatment, potentially contributing to the enhanced efficacy witnessed in our study. Additionally, a majority of our patients underwent a combined ICIs and chemotherapy regimen, in contrast to previous clinical trials that typically evaluated the efficacy of single-agent ICIs or dual-immunity therapy (Pouyiourou et al. 2023; Raghav, et al. 2022; Tanizaki et al. 2022). Recognizing the limitations often associated with standalone ICIs efficacy, our approach aimed to harness the potential synergy between chemotherapy and ICIs that may significantly contribute to the superior outcomes observed. Of note, a prior study of untreated unfavorable CUP patients receiving Site-Specific and Targeted Therapy based on NGS profiling reported a PFS of 5.2 months and an OS of 13.7 months (Hayashi et al. 2020). However, our study revealed that ICIs alone or in combination with chemotherapy yielded a notably longer median PFS of 9.7 months and a median OS of 23.73 months. Consequently, the combination of immunotherapy with chemotherapy emerges as a favorable and prudent therapeutic choice. Additionally, our study showed significantly higher ORR and a trend towards longer PFS and OS with ICIs + TP compared to ICIs + other, indicating that ICIs in combination with taxane-platinum based chemotherapy may have a better efficacy than ICIs combined with other chemotherapy in CUP patients. Nonetheless, the efficacy of ICIs has demonstrated success in a limited subset and biomarkers may be needed to identify the benefit population.

Some previous studies looking into the pathologic features of responders to ICI suggested that PD-L1 expression is a predictive biomarker for ICIs therapy. Despite the importance of understanding immune features across cancer types for their potential in ICIs responses, few studies have investigated the immune profile of CUP. Approximately one-third of CUP patients demonstrated tumors with a PD-L1 TPS of 1%, and antitumor immunity-related gene expression in CUP was similar to that in ICI-responsive malignancies (Haratani et al. 2019; Raghav, et al. 2022; Tanizaki et al. 2022). The data obtained from available PD-L1 testing revealed a notably higher prevalence of elevated PD-L1 expression among our patients, inconsistent with previous findings. This discrepancy could be attributed to the small sample size and individual differences. However, benefit populations are often stratified by the positive PD-L1 expression with various thresholds. We adopted CPS ≥ 20 as cutoff based on a previous clinical trial (Samstein et al. 2019), observing an association between tumor cell PD-L1 expression levels and the ORR for ICIs, consistent with previous studies conducted in CUP patients (Raghav, et al. 2022; Tanizaki et al. 2022). However, our survival data remain immature and longer follow-up is still needed to confirm the predictive value of PD-L1 for ICIs in patients with CUP.

The independent predictive value of TMB in ICIs has been previously demonstrated in several entities. Based on findings from the KEYNOTE-158 study associating TMB with improved response and survival to ICI across diverse solid tumors, the FDA approved pembrolizumab for individuals with high TMB, irrespective of the cancer's tissue of origin, thereby including CUP (Marabelle et al. 2020). Several studies analyzing the genomic profile of CUP reported that about 10—20% of cases harbor high TMB levels depending on TMB threshold applied (Gatalica et al. 2018; Pouyiourou et al. 2023; Gay, et al. 2017; Krämer, et al. 2018; Bochtler et al. 2022). Likewise, a significant minority of CUPs had high TMB in our study. Among CUP patients with high TMB, ICIs yielded better efficacy and induced a durable benefit, consistent with prior studies in CUP patients (Pouyiourou et al. 2023; Tanizaki et al. 2022). Our findings suggest that TMB might serve as a valuable predictive marker for ICIs in the context of CUP, thereby supplementing clinical trial evidence. However, to determine the predictive value of TMB for ICIs in CUP, prospective data involving larger cohorts of treatment efficacy and biomarker reliability are needed.

Prior studies have shown that KRAS mutations in malignancies may promote cellular proliferation, differentiation, and resistance to apoptosis through multiple downstream activation pathways (e.g., MAPK pathway, PI3K-AKT-mTOR pathway, etc.), which may lead to poorer prognosis. In addition, KRAS alterations may promote recruitment of immunosuppressive cells and downregulate PD-L1 expression, resulting in changes in tumour microenvironment (Parikh et al. 2022). We found that patients with KRAS alterations in our cohort did not significantly benefit from ICI treatment, and this alteration independently associated with inferior PFS and OS. This result is consistent with previous studies in CUP and non-CUP patients (Bochtler et al. 2020; Ricciuti et al. 2022), and may be attributed to the worse prognosis caused by KRAS mutations itself as well as the poorer response to ICIs due to changes in tumour microenvironment. However, the association of KRAS alterations and response to ICIs in CUP patients needs to be further validated in larger prospective clinical trials. As of the present, several KRAS inhibitors have been approved by FDA (Isermann et al. 2025). Therefore, the combination of KRAS inhibitors with ICIs could be considered in the future to increase the efficacy of ICIs in CUP patients with KRAS alterations.

In previous studies, the application of ICIs in CUP patients had predominantly focused on monotherapy or dual-immunotherapy combinations. However, in our study, the majority of patients received ICIs in combination with chemotherapy. Our results demonstrate that ICIs combined with chemotherapy may achieve favorable outcomes in CUP patients, with longer progression-free survival (PFS) and overall survival (OS) compared to data from prior studies. Therefore, our study provides preliminary clinical evidence for the use of ICIs in combination with chemotherapy in CUP patients and offers important insights for future research and clinical practice.

The limitations of this study lie in its retrospective nature and the heterogeneity in baseline characteristics and treatment factors, such as the uneven distribution of treatment lines in different groups, which could introduce potential bias. Furthermore, only 31 out of the 124 patients underwent PD-L1 detection, 26 conducted NGS and 25 had TMB assessment, in which only 5 patients were stratified to be TMB-H. The sample sizes of biomarker analyses were relatively small, which may have introduced potential selection bias and compromised statistical power, thereby limiting the generalizability of our findings. The main strength of the present study was that it analyzed the efficacy and safety of ICIs in 124 patients with CUP, offering valuable supplementary insights to clinical trials. Nevertheless, further prospective clinical trials involving a larger cohort of CUP patients are imperative to confirm these findings. Moreover, the inclusion of more genomic information is needed in the future.

In conclusion, ICIs showed encouraging efficacy with an acceptable safety profile in CUP patients, suggesting its potential as an additional therapeutic option for this population. Identifying predictive markers for ICIs response remains essential to enhance therapeutic strategies in CUP management.

## Supplementary Information

Below is the link to the electronic supplementary material.

Supplementary file1 (PDF 363 KB)

Supplementary file2 (PDF 877 KB)

Supplementary file3 (DOCX 13 KB)

Supplementary file4 (DOCX 13 KB)

Supplementary file5 (DOCX 17 KB)

Supplementary file6 (DOCX 14 KB)

## Data Availability

No datasets were generated or analysed during the current study.
