# Supplementary figures and images for: Clinical outcome and genomic biomarkers of immune checkpoint inhibitor-based therapies for cancer of unknown primary: a multicenter, real-world study
Source: J Cancer Res Clin Oncol. 2025 Jul 12;151(7):213. doi: 10.1007/s00432-025-06261-3 (PMC12255553; doi:10.1007/s00432-025-06261-3)

Fig. S1

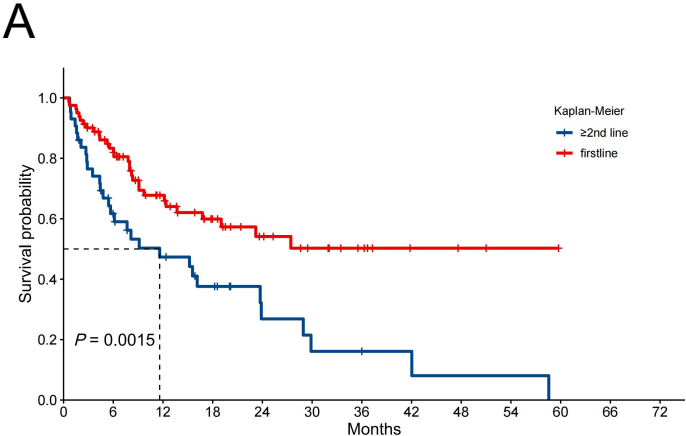

No. at risk:

|   |    |    |    |    |    |    |   |   |   |   |   |   |   |
|---|----|----|----|----|----|----|---|---|---|---|---|---|---|
| — | 43 | 23 | 16 | 11 | 5  | 3  | 3 | 2 | 1 | 1 | 0 | 0 | 0 |
| — | 81 | 60 | 37 | 24 | 16 | 11 | 7 | 3 | 2 | 1 | 0 | 0 | 0 |

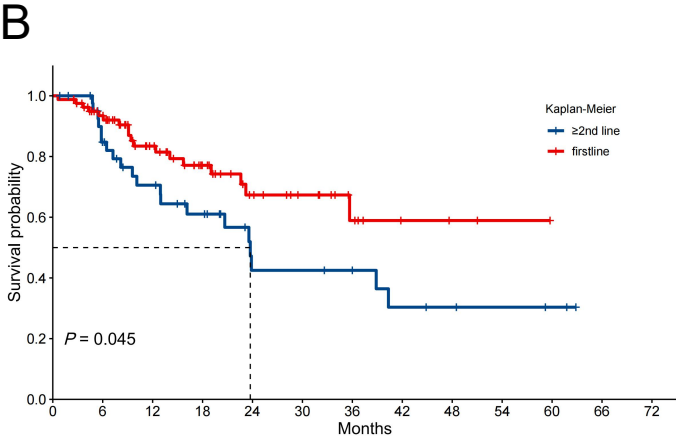

No. at risk:

|   |    |    |    |    |    |    |   |   |   |   |   |   |   |
|---|----|----|----|----|----|----|---|---|---|---|---|---|---|
| — | 43 | 32 | 24 | 18 | 9  | 9  | 8 | 5 | 4 | 3 | 2 | 0 | 0 |
| — | 81 | 66 | 43 | 29 | 18 | 13 | 7 | 3 | 2 | 1 | 0 | 0 | 0 |

Supplement: Supplementary file 1 — Supplementary file1 (PDF 363 KB) [file 432_2025_6261_MOESM1_ESM.pdf]

Fig. S2

A

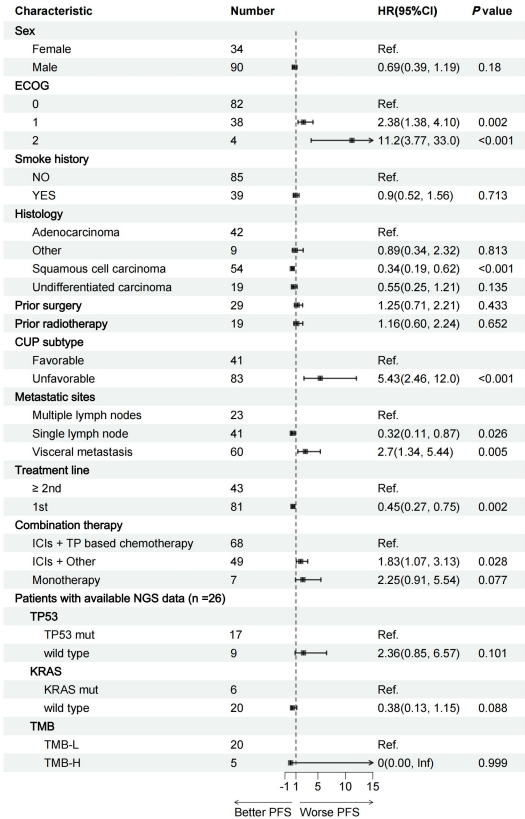

B

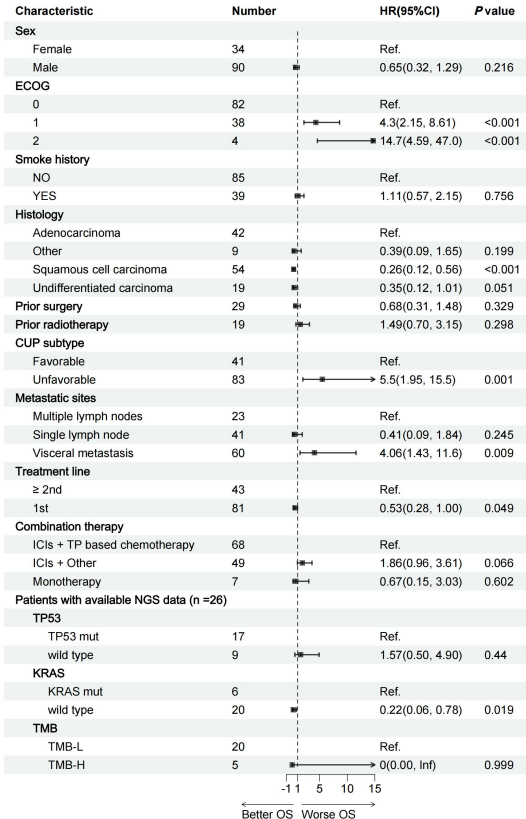

Supplement: Supplementary file 2 — Supplementary file2 (PDF 877 KB) [file 432_2025_6261_MOESM2_ESM.pdf]
