# Supplementary material for: Clinical outcome and genomic biomarkers of immune checkpoint inhibitor-based therapies for cancer of unknown primary: a multicenter, real-world study
Source: J Cancer Res Clin Oncol. 2025 Jul 12;151(7):213. doi: 10.1007/s00432-025-06261-3 (PMC12255553; doi:10.1007/s00432-025-06261-3)
Supplement: Supplementary file 3 — Supplementary file3 (DOCX 13 KB) [file 432_2025_6261_MOESM3_ESM.docx]

|  | First-line treatment  N(%) | ≥ 2nd line treatment  N(%) | Total, N(%) |
| --- | --- | --- | --- |
| TP based chemotherapy | 58 (72.50) | 10 (27.03) | 68 (58.12) |
| FOLFOX/FOLFIRINOX/FOLDOXIRI | 9 (11.25) | 1 (2.70) | 10 (8.55) |
| Platinum-based therapies | 4 (5.00) | 7 (18.92) | 11 (9.40) |
| Anti-angiogenic targeted therapy | 4 (5.00) | 8 (21.62) | 12 (10.26) |
| Capecitabine/S-1/5-FU | 2 (2.50) | 6 (16.22) | 8 (6.84) |
| XELOX | 2 (2.50) | 1 (2.70) | 3 (2.56) |
| Nab-paclitaxel+capecitabine | 0 (0) | 2 (5.41) | 2 (1.71) |
| Pemetrexed + carboplatin | 1 (1.25) | 0 (0) | 1 (0.85) |
| Nab-paclitaxel | 0 (0) | 1 (2.70) | 1 (0.85) |
| cellular therapy | 0 (0) | 1 (2.70) | 1 (0.85) |
| Total | 80 | 37 | 117 |

Table S1. The details of combination therapy
