# Supplementary material for: Clinical outcome and genomic biomarkers of immune checkpoint inhibitor-based therapies for cancer of unknown primary: a multicenter, real-world study
Source: J Cancer Res Clin Oncol. 2025 Jul 12;151(7):213. doi: 10.1007/s00432-025-06261-3 (PMC12255553; doi:10.1007/s00432-025-06261-3)
Supplement: Supplementary file 4 — Supplementary file4 (DOCX 13 KB) [file 432_2025_6261_MOESM4_ESM.docx]

**Table S2. Best overall response**

| Response | All  (N=124) | Favorable subset  (N=41) | Unfavorable subset  (N=83) |
| --- | --- | --- | --- |
| Objective response rate, N (%) | 69(55.65) | 33(80.49) | 36(43.37) |
| Best overall response, N (%) |  |  |  |
| Complete response | 19(15.32) | 15(36.59) | 4(4.82) |
| Partial response | 50(40.32) | 18(43.90) | 32(38.55) |
| Stable disease | 33(26.61) | 5(12.20) | 28(33.73) |
| Progressive disease | 15(12.10) | 2(4.88) | 13(15.66) |
| Nonevaluable | 7(5.65) | 1(2.44) | 6(7.23) |
| Disease control rate, N (%) | 102(82.26) | 38(92.68) | 64(77.11) |
