# Supplementary material for: Clinical outcome and genomic biomarkers of immune checkpoint inhibitor-based therapies for cancer of unknown primary: a multicenter, real-world study
Source: J Cancer Res Clin Oncol. 2025 Jul 12;151(7):213. doi: 10.1007/s00432-025-06261-3 (PMC12255553; doi:10.1007/s00432-025-06261-3)
Supplement: Supplementary file 5 — Supplementary file5 (DOCX 17 KB) [file 432_2025_6261_MOESM5_ESM.docx]

Table S3. Disease characteristics of 31 patients with available PD-L1 detection results.

|  | Total  (N=31) ,N(%) | CPS＜20  (N=16) ,N(%) | CPS≥20  (N=15) ,N(%) | *P* value |
| --- | --- | --- | --- | --- |
| Age |  |  |  |  |
| Median (range) | 56(37-75) | 55(37-72) | 58(39-75) |  |
| Sex |  |  |  |  |
| Female | 10 (32.26) | 7 (43.75) | 3 (20.00) | 0.2524 |
| Male | 21 (67.74) | 9 (56.25) | 12 (80.00) |  |
| ECOG performance status |  |  |  |  |
| 0 | 24 (77.42) | 13 (81.25) | 11 (73.33) | 0.6851 |
| 1 | 7 (22.58) | 3 (18.75) | 4 (26.67) |  |
| Smoking history | 11 (35.48) | 3 (18.75) | 8 (53.33) | 0.0659 |
| Histology |  |  |  |  |
| Adenocarcinoma | 8 (25.81) | 6 (37.50) | 2 (13.33) | 0.3764 |
| Squamous cell carcinoma | 14 (45.16) | 5 (31.25) | 9 (60.00) |  |
| Undifferentiated carcinoma | 6 (19.35) | 3 (18.75) | 3 (20.00) |  |
| Other | 3 (9.68) | 2 (12.50) | 1 (6.67) |  |
| CUP subtype |  |  |  |  |
| Favorable subset | 12 (38.71) | 3 (18.75) | 9 (60.00) | 0.0469 |
| Unfavorable subset | 19 (61.29) | 13 (81.25) | 6 (40.00) |  |
| Visceral metastasis site |  |  |  |  |
| Peritoneal or omental implantation | 3 (9.68) | 2 (12.50) | 1 (6.67) | 1 |
| Adrenal gland | 2 (6.45) | 1 (6.25) | 1 (6.67) | 1 |
| Liver | 2 (6.45) | 1 (6.25) | 1 (6.67) | 1 |
| Lung | 3 (9.68) | 2 (12.50) | 1 (6.67) | 1 |
| Bone | 3 (9.68) | 2 (12.50) | 1 (6.67) | 1 |
| Prior radiotherapy | 4 (12.90) | 3 (18.75) | 1 (6.67) | 0.5996 |
| Prior surgery | 10 (32.26) | 5 (31.25) | 5 (33.33) | 1 |
| Treatment line |  |  |  |  |
| 1st | 24 (77.42) | 10 (62.50) | 14 (93.33) | 0.0937 |
| 2nd | 3 (9.68) | 2 (12.50) | 1 (6.67) |  |
| ≥ 3rd | 4 (12.90) | 4 (25.00) | 0 (0.00) |  |
| Regimen |  |  |  |  |
| Monotherapy | 1 (3.23) | 0 (0.00) | 1 (6.67) | 0.4839 |
| Combination therapy | 30 (96.77) | 16 (100.00) | 14 (93.33) |  |
