# Supplementary material for: Clinical outcome and genomic biomarkers of immune checkpoint inhibitor-based therapies for cancer of unknown primary: a multicenter, real-world study
Source: J Cancer Res Clin Oncol. 2025 Jul 12;151(7):213. doi: 10.1007/s00432-025-06261-3 (PMC12255553; doi:10.1007/s00432-025-06261-3)
Supplement: Supplementary file 6 — Supplementary file6 (DOCX 14 KB) [file 432_2025_6261_MOESM6_ESM.docx]

Table S4. Disease characteristics of 26 patients with available next-generation sequencing data.

|  | Total  (N=26) ,N(%) |
| --- | --- |
| Age |  |
| Median (range) | 60 (40-72) |
| Sex |  |
| Female | 8 (30.77) |
| Male | 18 (69.23) |
| ECOG performance status |  |
| 0 | 12 (46.15) |
| 1 | 13 (50.00) |
| 2 | 1 (3.85) |
| Smoking history | 7 (26.92) |
| Histology |  |
| Adenocarcinoma | 17 (65.38) |
| Squamous cell carcinoma | 2 (7.69) |
| Undifferentiated carcinoma | 5 (19.23) |
| Other | 2 (7.69) |
| CUP subtype |  |
| Favorable subset | 3 (11.54) |
| Unfavorable subset | 23 (88.46) |
| PD-L1 expression |  |
| CPS＜20 | 6 (23.08 ) |
| CPS≥20 | 4 (15.38) |
| NA | 16 (61.54) |
| Visceral metastasis site |  |
| Peritoneal or omental implantation | 10 (38.46) |
| Adrenal gland | 4 (15.38) |
| Liver | 3 (11.54) |
| Lung | 5 (19.23) |
| Bone | 8 (30.77) |
| Prior radiotherapy | 2 (7.69) |
| Prior surgery | 6 (23.08) |
| Treatment line |  |
| 1st | 17 (65.38) |
| 2nd | 6 (23.08) |
| ≥ 3rd | 3 (11.54) |
| Regimen |  |
| ICIs + TP based chemotherapy | 12 (46.15) |
| ICIs + Other | 13 (50.00) |
| Monotherapy | 1 (3.85) |
